# Successful medical management of a case of Austrian syndrome—an uncommon entity in the modern antibiotic era: a case report

**DOI:** 10.1186/s13104-017-2801-8

**Published:** 2017-09-06

**Authors:** Muhammad Abdur Rahim, Shahana Zaman, Hasna Fahmima Haque, Samira Rahat Afroze, Khwaja Nazim Uddin

**Affiliations:** 1 Bangladesh Institute of Research and Rehabilitation in Diabetes, Endocrine and Metabolic Disorders (BIRDEM) General Hospital, 122, Kazi Nazrul Islam Avenue, Dhaka, Bangladesh; 2 National Institute of Cardiovascular Diseases (NICVD), Dhaka, Bangladesh

**Keywords:** Austrian syndrome, Infective endocarditis, Meningitis, Pneumonia, *Streptococcus pneumoniae*

## Abstract

**Background:**

Austrian syndrome—the combination of meningitis, pneumonia and infective endocarditis due to *Streptococcus pneumoniae* infection, is a rare entity. In literature only a few hundreds of cases are reported but surprisingly we did not find any report on Austrian syndrome in or from Bangladesh.

**Case presentation:**

We report the case history of a middle aged Bangladeshi diabetic man, who had fever, cough, shortness of breath and altered mentation. He had tachycardia, bi-basal lung crepitations, new cardiac murmurs and meningism. Diagnostic work-up revealed Austrian syndrome. Because of the rarity of the condition, this case is reported.

**Conclusion:**

A case of pneumococcal pneumonia or meningitis should raise suspicion of concomitant endocarditis and Austrian syndrome, specially if there is heart failure, as early recognition and treatment may appear life-saving.

**Electronic supplementary material:**

The online version of this article (doi:10.1186/s13104-017-2801-8) contains supplementary material, which is available to authorized users.

## Background

Austrian syndrome is the rare combination of meningitis, infective endocarditis and pneumonia; all occurring due to infection by *Streptococcus pneumoniae*. Debilitated middle-aged alcoholic men are the usual sufferers [1]. Native aortic valve involvement is the most common cardiac lesion, mitral valve involvement occurs in one-third of the cases [2] and quadruple valve involvement is also possible [3]. Austrian syndrome occurs in 14% of *S. pneumoniae* endocarditis cases and carries a high mortality [2]. Here, we report a case of Austrian syndrome occurring in a middle aged Bangladeshi diabetic man, successfully treated by medical management. To our knowledge, this is the first case of Austrian syndrome being reported from Bangladesh.

## Case presentation

A 48-year-old Bangladeshi gentleman was brought to the emergency room because of breathlessness and disorientation. He had been suffering from fever, cough and sputum production over the preceding 5 days. He had been receiving amoxicillin (orally 500 mg three times a day) and paracetamol (500 mg four times a day) without much benefit. He did not have significant medical history of note, except diabetes mellitus and was on vildagliptin–metformin combination (vildagliptin 50 mg and metformin 500 mg orally once daily).

He was febrile (temperature 38.9 °C), had altered conscious level (Glasgow Coma Scale E3V4M4, 11/15), low oxygen saturation (87% in room air), tachycardia (pulse 110/min), tachypnea (respiratory rate 38/min), normal blood pressure (115/75 mmHg), bi-basal crepitations and signs of meningeal irritation without any rash. Ocular fundi looked normal.

Random blood glucose was 8.9 mmol/L. Electrocardiography revealed sinus tachycardia, chest X-ray showed pulmonary oedema, computed tomography of head was normal. Arterial blood gas analysis showed type I respiratory failure. He was shifted to intensive care unit.

He had neutrophil leukocytosis (total white cell count 22,300/mm^3^, neutrophil 83%), high erythrocyte sedimentation rate (56 mm in 1st hour) and C-reactive protein (64 mg/L). Cerebrospinal fluid (CSF) was cloudy with high opening pressure, high protein (140.6 mg/dL, reference range 15–45 mg/dL) and low glucose (3.1 mmol/L, reference range 3.3–4.5 mmol/L) content, had high white cell counts with predominant neutrophils (1290 cells/mm^3^, polymorphs 90%). No micro-organism was identified in Gram-stain or acid fast bacilli (AFB) staining of CSF or cultures. Bacterial antigen test of CSF (done by Latex test to detect *Streptococcus* group B, *H. influenza* type B, *S. pneumoniae*, *N. meningitides* ABCY W135 and *E. coli* K1 antigens, Wellcogen^™^ Bacterial Antigen Kit, Remel Europe Ltd., UK) was positive for pneumococcus. Blood culture did not reveal any organism. Sputum microscopy revealed Gram positive cocci in long chains but culture did not reveal any organism. Urine routine examination showed red cells (5–8/high power field) and protein (+).

Treatment consisted of ceftriaxone (2 g intravenously every 12 h), moxifloxacin (400 mg intravenously once daily), paracetamol, frusemide and insulin along with other supportive measures including oxygen. He became afebrile on fourth day and was transferred to general medical ward.

**Fig. 1:**
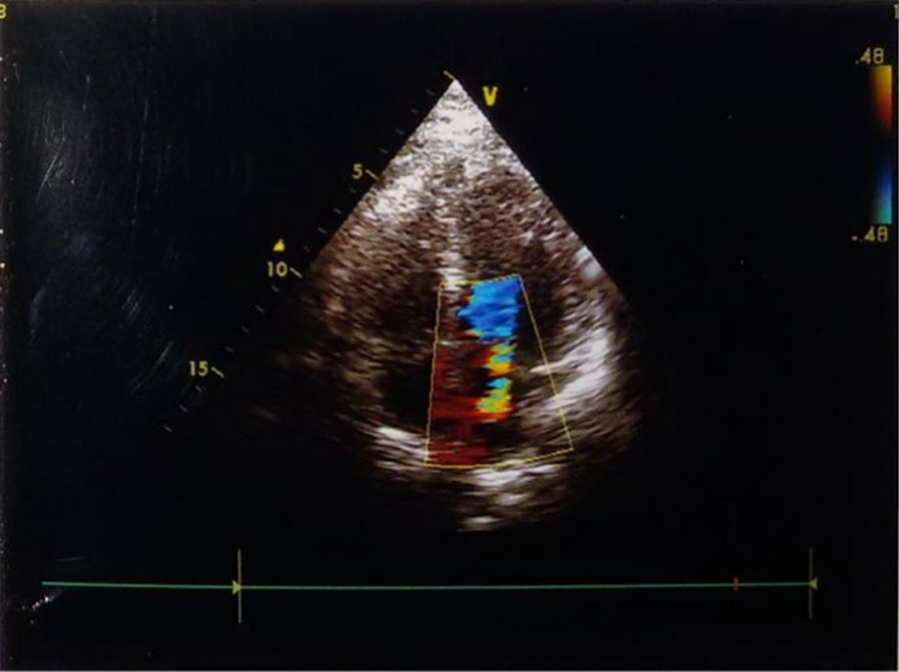
Color Doppler echocardiography parasternal long axis view showing moderate mitral regurgitation

**Fig. 2:**
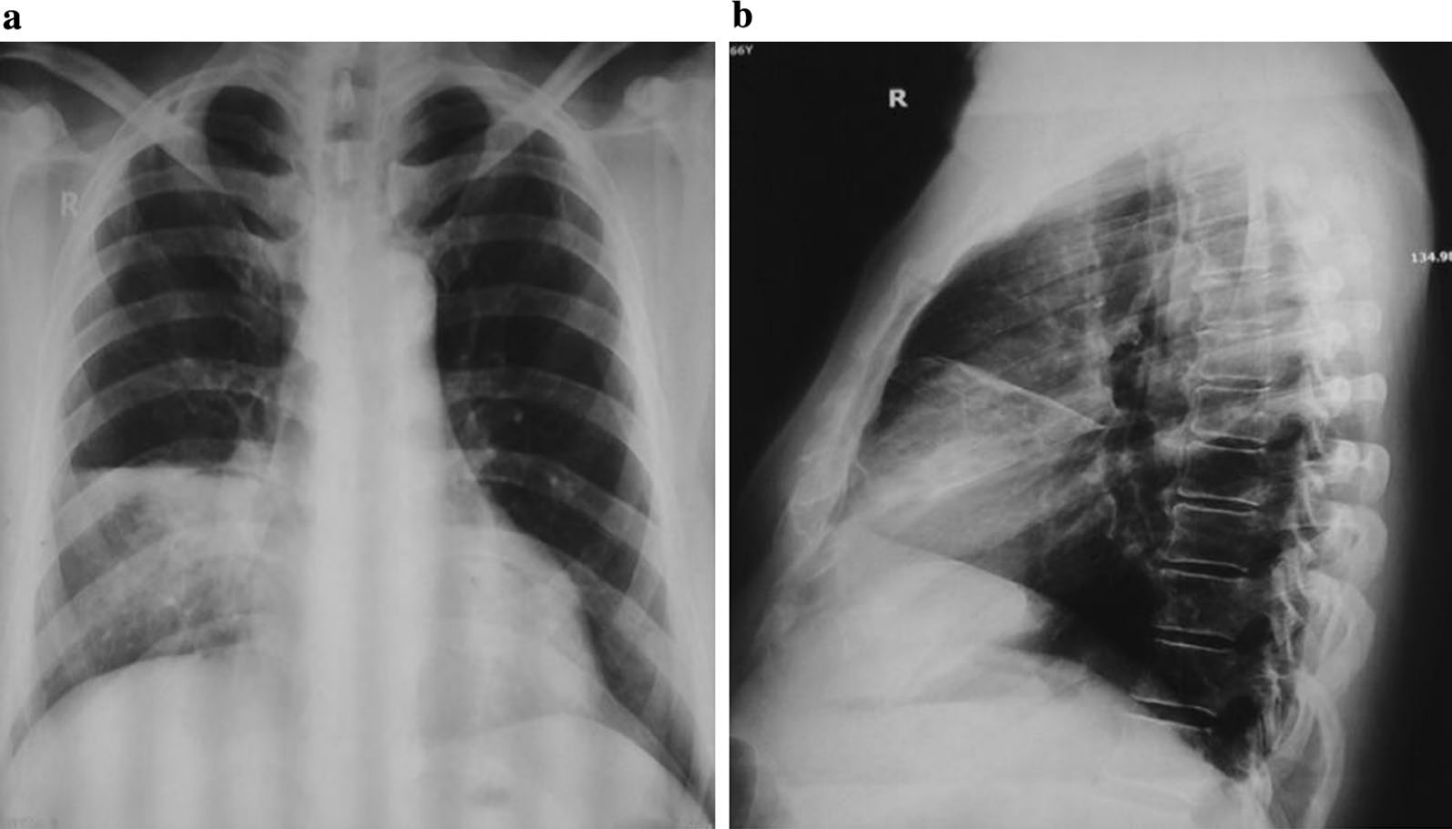
Chest X-ray (**a**) postero-anterior and (**b**) right lateral view showing right middle lobe consolidation

His assessment in ward showed a new regurgitant murmur in mitral area that radiated to left axilla. Transthoracic echocardiography revealed moderate mitral regurgitation (Fig. 1) but there was no vegetation. Trans-oesophageal echocardiography could not be done. Repeat chest X-ray revealed right middle lobe consolidation (Fig. 2a, b). Depending upon clinical manifestation, imaging, echocardiography, sputum, CSF and urine examination findings, he was diagnosed as having Austrian syndrome. He completed 4 weeks of intravenous antibiotic treatment, follow-up echocardiography after 6 weeks revealed trivial mitral regurgitation (Additional file 1).

## Discussion

*Streptococcus pneumoniae* remains as the predominant organism for community acquired pneumonia and meningitis, but its role in causation of infective endocarditis has fallen to less than 3% cases from 15–30% in the pre-penicillin era [4, 5]. It is not uncommon that, during an episode of invasive pneumococcal infection like meningitis; the possibility of valvular involvement is easily overlooked in absence of peripheral stigmata of infective endocarditis [6, 7] resulting in late diagnosis of endocarditis [1, 8].

Identification of the causative organism in blood cultures and demonstration of valve vegetation(s) on echocardiography are important in establishing a diagnosis of infective endocarditis. Five to seven percent of infective endocarditis cases may be culture negative and this may be increased, if patients had taken antibiotic. Trans-oesophageal echocardiography has higher sensitivity in identification of small vegetations in infective endocarditis [9]. Antibiotic alone may efficiently clear-off vegetations in a small number of cases and a later performed echocardiography may fail to detect vegetations in such cases [10].

In our case, we could not identify causative organism in blood, CSF or sputum cultures, but pneumococcal antigen was positive in CSF and sputum microscopy finding was in favour of presence of *S. pneumoniae*; history of prior antibiotic intake well explains all these things. Our hospital did not have the facility to do trans-oesophageal echocardiography and as the patient was recovering satisfactorily, we did not pursue for a trans-oesophageal echocardiography from another center. Improvement of mitral regurgitation on follow-up echocardiography supports that this regurgitation was an acute insult on the mitral valve.

## Conclusions

Even in the modern antibiotic era, Austrian syndrome has a high mortality. Aggressive medical or combined medical and surgical approach can be life-saving. Early recognition of the condition is of paramount importance for selection of an appropriate treatment strategy and may determine outcome. So, a high index of clinical suspicion is warranted.

## Additional file

**Additional file 1.** Timelines of events.

## Abbreviations

CSF: cerebrospinal fluid
AFB: acid fast bacilli
