# Supplementary material for: Successful medical management of a case of Austrian syndrome—an uncommon entity in the modern antibiotic era: a case report
Source: BMC Res Notes. 2017 Sep 6;10:456. doi: 10.1186/s13104-017-2801-8 (PMC5588715; doi:10.1186/s13104-017-2801-8)
Supplement: Supplementary file 1 — Additional file 1. Timelines of events. [file 13104_2017_2801_MOESM1_ESM.pdf]

Timelines of events

March 11, 2017	Patient developed fever, cough
March 12, 2016	Amoxicillin started OTC
March 16, 2017	Admission in ICU with SOB, altered mentation
March 20, 2017	Became afebrile, shift to ward
March 21, 2017	TTE done, MR detected, CXR-pneumonia
March 30, 2017	Discharged with advice to continue IV ceftriaxone
April 30, 2017	Repeat ECHO – improved, trivial MR
